# Comment on “Risk factors associated with chronic cutaneous graft-versus-host disease following hematopoietic stem cell transplantation: a pediatric cohort”^☆^

**DOI:** 10.1016/j.abd.2026.501456

**Published:** 2026-08-28

**Authors:** Neeraj Singh, Monika Srivastav

**Affiliations:** a Dr. D. Y. Patil Medical College Hospital and Research Centre, Dr. D. Y. Patil Vidyapeeth (Deemed-to-be-University), Pimpri, Pune, Maharashtra, India; b Dr. D. Y. Patil Dental College and Hospital, Dr. D. Y. Patil Vidyapeeth (Deemed-to-be-University), Pimpri, Pune, Maharashtra, India

Dear Editor,

We read with considerable interest the study by Carvajal et al., which evaluates risk determinants of chronic cutaneous Graft-Versus-Host Disease (ccGVHD) in a pediatric Hematopoietic Stem Cell Transplantation (HSCT) cohort.^1^ The extended follow-up duration, dermatology-confirmed diagnoses aligned with National Institutes of Health (NIH) criteria, and survival-based modeling provide a structured framework for examining temporal risk emergence in this clinically heterogeneous condition. The reported incidence of 17.3% and median onset at 8 months offer clinically relevant benchmarks for surveillance strategies. However, certain aspects merit further discussion.

A key interpretive consideration arises from the interdependence between graft source, donor type, and conditioning exposure within the cohort. Bone marrow grafts demonstrated the strongest independent association with ccGVHD (HR = 7.28), yet this variable is closely coupled with related donor status, where 96.4% of related transplants utilized bone marrow compared to predominant umbilical cord blood use in unrelated settings. This clustering introduces structural collinearity that may amplify the apparent independent effect of graft source within multivariate modeling.^2^ A stratified or interaction-based analysis separating graft source from donor relationship would clarify whether the observed hazard reflects intrinsic graft immunobiology or underlying transplant selection patterns.

An additional layer of complexity emerges from the temporal positioning of acute GVHD relative to ccGVHD development. While prior or concomitant acute GVHD demonstrated significance in univariate analysis (HR = 2.76), its exclusion from the final multivariate model limits interpretive continuity regarding immunologic progression. Given the observed 80.8% prevalence of acute GVHD among ccGVHD cases, incorporation as a time-dependent covariate could better delineate whether it functions as a mediator, an early clinical marker, or a confounder linked to conditioning intensity and graft characteristics.^3^

The identification of Total Body Irradiation (TBI) as an independent risk factor (HR = 3.53) also warrants contextualization within conditioning heterogeneity. TBI-based regimens are often employed in higher-risk malignancies or specific transplant protocols, potentially embedding disease severity and prior treatment exposure into the observed association.^4^, ^5^ Adjustment for underlying indication or regimen intensity would strengthen causal inference regarding TBI-driven tissue injury and downstream fibrotic cutaneous manifestations.

We commend the authors for providing pediatric-specific, clinically actionable data in an area with limited dedicated evidence. Future studies incorporating multicenter cohorts with harmonized transplant protocols, along with time-dependent modeling of immune events and stratified analyses disentangling graft source and donor characteristics, would further refine risk prediction and support the development of tailored surveillance and prevention strategies for ccGVHD.

## ORCID ID

Monika Srivastav: 0009-0005-4778-5962

## Declaration of generative artificial intelligence (AI)

During the preparation of this work, the author(s) used ChatGPT and Grammarly in order to improve language clarity and grammatical accuracy. After using these tools, the author(s) reviewed and edited the content as needed and take full responsibility for the content of the publication.

## Financial support

None declared.

## Authors’ contributions

Neeraj Singh: Approval of the final version of the manuscript; critical literature review; data collection, analysis and interpretation; effective participation in research orientation; intellectual participation in propaedeutic and/or therapeutic management of studied cases; manuscript critical review; preparation and writing of the manuscript; statistical analysis; study conception and planning.

Monika Srivastav: Approval of the final version of the manuscript; critical literature review; preparation and writing of the manuscript.

## Research data availability

Does not apply.

## Conflicts of interest

None declared.
